# Metals and Neuronal Metal Binding Proteins Implicated in Alzheimer's Disease

**DOI:** 10.1155/2016/9812178

**Published:** 2016-01-10

**Authors:** Joana S. Cristóvão, Renata Santos, Cláudio M. Gomes

**Affiliations:** ^1^Faculdade de Ciências Universidade de Lisboa, Biosystems and Integrative Sciences Institute and Department of Chemistry and Biochemistry, Universidade de Lisboa, Campo Grande, 1749-016 Lisboa, Portugal; ^2^Development of the Nervous System, Institut de Biologie de l'Ecole Normale Supérieure (IBENS), INSERM U1024, CNRS UMR8197, 46 rue d'Ulm, 75005 Paris, France

## Abstract

Alzheimer's disease (AD) is the most prevalent age-related dementia affecting millions of people worldwide. Its main pathological hallmark feature is the formation of insoluble protein deposits of amyloid-*β* and hyperphosphorylated tau protein into extracellular plaques and intracellular neurofibrillary tangles, respectively. Many of the mechanistic details of this process remain unknown, but a well-established consequence of protein aggregation is synapse dysfunction and neuronal loss in the AD brain. Different pathways including mitochondrial dysfunction, oxidative stress, inflammation, and metal metabolism have been suggested to be implicated in this process. In particular, a body of evidence suggests that neuronal metal ions such as copper, zinc, and iron play important roles in brain function in health and disease states and altered homeostasis and distribution as a common feature across different neurodegenerative diseases and aging. In this focused review, we overview neuronal proteins that are involved in AD and whose metal binding properties may underlie important biochemical and regulatory processes occurring in the brain during the AD pathophysiological process.

## 1. Alzheimer's Disease: Hallmark Amyloid Aggregation and Neuronal Dysfunction

Alzheimer's disease (AD) is a progressive neurodegenerative disorder characterized by cognitive decline. The neuropathology hallmarks are gross atrophy of the cortex and hippocampus, and the accumulation of amyloid-beta (A*β*) into senile plaques and of hyperphosphorylated tau into neurofibrillary tangles. The deposition of A*β* and hyperphosphorylated tau aggregates in the human brain occurs in opposite directions with an orderly neuroanatomical pattern. Amyloid plaques first appear in the neocortex and slowly progress through the striatum, the basal cholinergic nuclei, the brain stem, and finally the cerebellum [1]. The deposition of tangles begins in the brain stem and progresses towards the neocortex [2]. Thus, the common presence of amyloid plaques and tau neurofibrillary tangles in the cortex only happens at late stages of the disease.

AD is heterogeneous and multifactorial with sporadic and familial forms [3–6]. The large majority of patients have the sporadic form or late onset dementia (later than 65 years). The few remaining patients have the familial form with early onset dementia (around 30 years to 65 years) and may present different symptoms. These patients have mutations in one of three genes encoding proteins essential for A*β* formation: the amyloid precursor protein (APP) and presenilins 1 and 2 (PSEN1/2) [7–10]. Presenilins are components of catalytic subunit of *γ*-secretase multicomplex, responsible for the cleavage of APP and formation of A*β*. The origin of the sporadic form is complex involving multiple genetic and environmental risk factors, for example, the presence of apolipoprotein E-*ε*4 allele, mitochondrial dysfunction, head injury, or a compromised brain blood barrier [3, 11]. Despite the fact that AD is the most common form of dementia of the elderly and affects millions of people worldwide, the exact cause of this disorder is still unknown. The genetic evidence obtained from the rare familial form of AD supports the hypothesis that the accumulation of A*β* plaques is at the origin of the disease. This is the foundation for the amyloid-*β* cascade hypothesis [12] which has been the central theory in AD research for the last three decades. According to this hypothesis, the deposition of A*β* is the initial event and it is sufficient to trigger the cascade of pathological and clinical changes in AD, which are the formation of senile plaques and neurofibrillary tangles and subsequent neuronal death, vascular damage, and dementia [12]. Although senile plaque deposition is an early event in the disease, as observed in postmortem human brains [1], plaque accumulation in the brain does not correlate with dementia [13] implying that other mechanisms are associated with neurodegeneration. Notably, therapies designed until now that aimed at targeting amyloid plaques and APP proved to be largely unsuccessful. An increasing amount of data challenges the amyloid-*β* cascade hypothesis.

Therefore, efforts to integrate the other pathogenic features of AD and multiple etiology pathways into a more global model are now needed. During the course of AD, tau is hyperphosphorylated and accumulates in the somatodendritic compartment as paired helical filaments and straight filaments [14]. In neurons, tau is the major microtubule associated protein and stabilizes its structure. Tau interacts with tubulin promoting its assembly into microtubules. The level of phosphorylation regulates the activity of tau and hyperphosphorylation suppresses its microtubule assembly activity. In addition, hyperphosphorylated tau sequesters normal tau and other microtubule associated proteins that further contribute to microtubule disassembly [15]. Therefore, the abnormal phosphorylation of tau results in loss of normal function and gain of toxic function in the AD brain. The formation of neurofibrillary tangles does correlate with cognitive decline and with neuronal and synapse loss [13, 16].

Senile plaques are extracellular deposits composed mainly of amyloid peptides ranging from 39 to 43 amino acids, which are natural metabolites of APP generated by sequential cleavage by *β*-secretase and *γ*-secretase l [17]. The APP is a transmembrane protein necessary for neurogenesis, for neurite outgrowth and guidance, and for synapse formation and repair [18]. APP is processed in different ways through different enzymes leading to the formation of amyloidogenic and nonamyloidogenic precursors. The processing of APP results in the formation of soluble *α*- and *β*-secreted APP (sAPP) which is cleaved by *α*- and *β*-secretase, respectively. As a product in the nonamyloidogenic pathway, sAPP*α* promotes neuronal survival and neurite outgrowth, among other beneficial neuronal functions. Contrarily, sAPP*β* is not involved in the beneficial functions of sAPP*α*, participating in synapse pruning. A*β* is secreted through sequential APP cleavage by *β*- and *γ*-secretases, resulting in peptides that can range from 39 to 43 amino acids. The A*β* peptides are catabolized by multiple amyloid degrading enzymes, for example, neprilysin and insulin-degrading enzyme [19]. It is the imbalance between the production and clearance of A*β* that triggers its deposition as amyloid plaques. However, several studies suggest that A*β* has a physiological role in the synapses and its complete removal induces neuronal cell death [20–22]. In addition to the aggregates, A*β* is also present in soluble oligomeric forms in APP-transgenic mice and human diseased brains [20]. Compared to A*β* aggregates, the soluble oligomers are highly neurotoxic [23]. Therefore, it is possible that aggregation of A*β* into plaques is a neuroprotective mechanism that eliminates the toxic oligomeric forms [15].

The normal functions of synapses are impaired during the course of AD. Synapse loss correlates with dementia suggesting that it is important for disease progression and for the degeneration process [24]. Dense plaque deposition causes the surrounding neurites to bend and change trajectory, which can lead to changes in synapse signal transmission. Also, gliosis and oxidative stress are observed in the vicinity of plaques. During normal development of the brain, microglia are involved in synaptic pruning after birth and it is possible that in the diseased AD brain the recruitment of activated microglia around the plaques participates in the synapse loss [24]. In addition to aggregates, the oligomeric forms of A*β* obtained from cultured cells or from human AD brain disturb synapses and lead to cognition impairment in injected mice [25–27]. Comparably, evidence also shows that soluble forms of tau are toxic for synapses [28]. The molecular mechanisms that lead to synapse dysfunction and neuronal loss downstream of A*β* and tau are not completely identified but different pathways are implicated such as mitochondrial dysfunction, oxidative stress, inflammation, and dysregulation of metal homeostasis.

## 2. Metals and Metal Binding Proteins Implicated in AD

Metal ions play essential roles in the brain and there is solid evidence pointing to their homeostatic dysfunction across different neurodegenerative diseases (e.g., [29–31]). This includes the first row transition metals, iron, copper, and zinc and also calcium, whose homeostasis is important for neuronal function and during aging [32–34]. One major hypothesis for this cross talk, which has been put forward since a number of years and which has been elegantly reviewed in [35], proposes that AD is as much as a metallopathy as a proteinopathy. Indeed, age-related metal ion dysfunction altered levels of neuronal metal ions in AD-affected areas including accumulation in protein deposits, and the interplay between metal ions and AD pathological proteins indicates a close relationship between protein misfolding, aggregation, and metal ion homeostasis. In AD patients, it has been shown that Cu^2+^, Zn^2+^, and Fe^2+^ are found in the core and rims of senile plaques [36, 37] and colocalize with A*β* [38]. This has led to the suggestion that metal ion sequestration into plaques could lead to deficient distribution of these metals in the neighbouring regions [39]. Moreover, it is described that in AD patients Zn^2+^ is decreased in serum and blood but increased in the cerebrospinal fluid and neocortical tissue [40–42]. In addition, Zn^2+^, Cu^2+^, and Fe^2+^ are increased in the neuropil of AD patients [36, 43]. In agreement with a role of metal ions in pathology, molecules designed to chelate Zn^2+^ and Cu^2+^ from amyloid-beta aggregates [44, 45] were found to decrease A*β* deposits in mice models due to A*β* solubilisation [45]. Here, as a contribution for a broader molecular and biochemical analysis of protein-metal cross talks in neurodegeneration, we undertake an overview of proteins with metal binding properties which are implicated in AD (Table 1).

### 2.1. Amyloid-*β*

**Figure 1:**
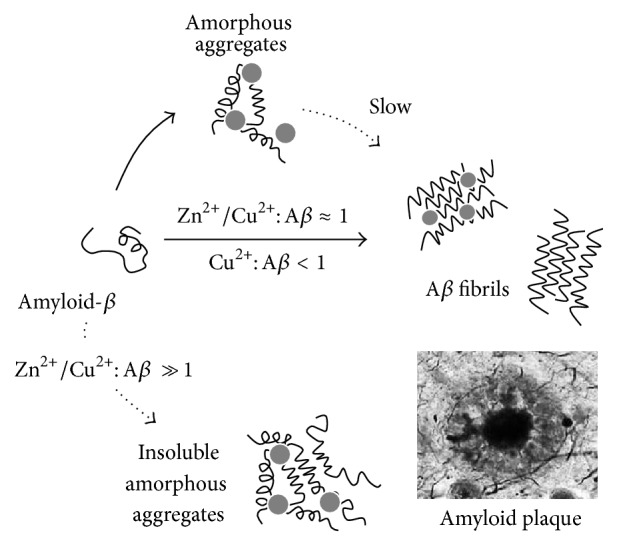
Modulation of amyloid-*β* aggregation by Cu^2+^ and Zn^2+^ binding. A*β* aggregation into fibrils is a complex pathway that involves multiple intermediate precursor species. The scheme is a simplification depicting direct effects of Cu^2+^ and Zn^2+^ on A*β* aggregation. Superstoichiometric levels of Cu^2+^ and Zn^2+^ (Zn^2+^/Cu^2+^: A*β* ≫ 1) result in insoluble and amorphous aggregates rather than organized fibrils, while equimolar Cu^2+^ and Zn^2+^ (Zn^2+^/Cu^2+^: A*β* ≈ 1) induce amorphous aggregates, which slowly convert to fibrils. At subequimolar Cu^2+^ levels (Cu^2+^: A*β* < 1), the kinetics of fibril formation are accelerated. The AD amyloid plaques, depicted in a representation at the bottom right corner of the figure, contain high levels of Zn (1055 *μ*M), Fe (940 *μ*M), and Cu (390 *μ*M), as reviewed in [35]. See text for details.

Metal ions have been acknowledged as important players of the pathological effects of A*β* aggregation in AD and have been considered as possible modulators of A*β* misfolding and aggregation due to their binding to the A*β* peptide [46–49] and to amyloid fibrils [50, 51]. Cu^2+^, Zn^2+^, and Fe^2+^ bind to A*β* influencing its aggregation pathway and are found in and nearby extracellular senile plaques [29, 36]. The binding of metal ions to A*β* invariably results in aggregation which may either be into amyloid fibers or into amorphous aggregates, depending on the metal ion, stoichiometry, and environmental conditions [49]. In spite of contradictory findings, there seems to be a consensus that (a) superstoichiometric levels of Cu^2+^ and Zn^2+^ result in insoluble and amorphous aggregates rather than organized fibrils [49, 52–55]; (b) equimolar Zn^2+^ and Cu^2+^ induce amorphous aggregates, which slowly convert to fibrils [56, 57]; and (c) at subequimolar Cu^2+^ levels, the kinetics of fibril formation are accelerated [52, 58, 59] (Figure 1). The observation that high levels of Zn^2+^ and Cu^2+^ seem to shift aggregation into oligomeric precursors rather than organized fibrils has important consequences in brain function, as these A*β* precursors are now known to be the neurotoxic self-propagating species causing neurodegeneration. Furthermore, Cu^2+^ and Fe^2+^ participate in ROS production causing oxidative stress and neuronal damage, thus being one of the causes that potentiate A*β* toxicity [60–62]. Indeed, the formation of H_2_O_2_ as a product of the interaction between A*β* and Cu^2+^ can generate hydroxyl radicals, which are related to AD pathology [63]. Superoxide has also been recently shown to be an intermediate of the reaction leading to the production of H_2_O_2_ by Cu^+^-A*β* and O_2_ [64]. Zinc and copper chelators inhibit A*β* plaque deposition in AD patients [44, 65, 66], further suggesting that amyloid pathology may arise from the dysregulation of these metal ions. Excess of iron increases A*β* production [67] and leads to the formation of annular protofibrils [68] and slows down the formation of ordered cross-*β* fibrils [69] towards the formation of shorter and less ordered aggregates [53, 69] which are potentially more toxic.

### 2.2. Tau

**Figure 2:**
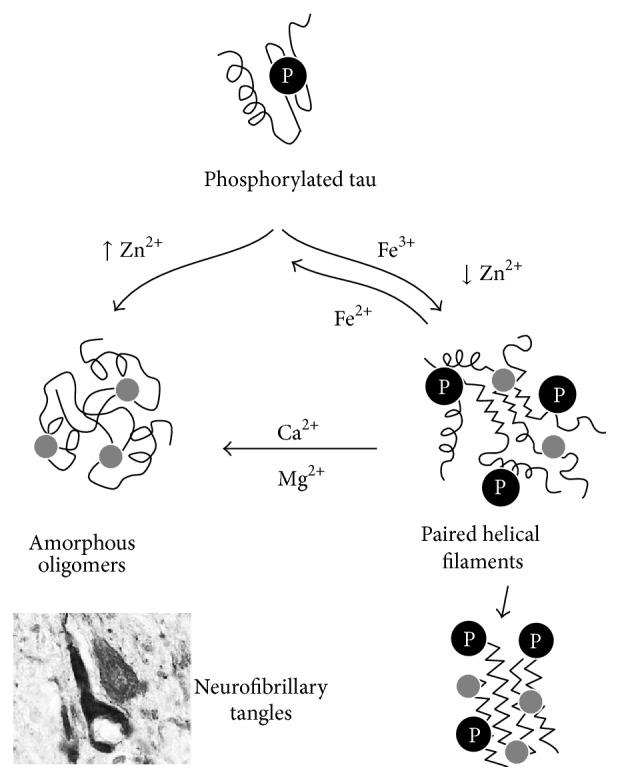
Modulation of tau aggregation by metal ions. Hyperphosphorylated (P) tau undergoes aggregation, which is influenced by metal ion binding. Tau phosphorylation facilitates Fe^3+^ binding that promotes the formation of paired helical filaments (PHF) and further tau fibrillation. The reduction of Fe^3+^ to Fe^2+^ reverts PHF formation. Zn^2+^ binding at high ratios promotes the formation of amorphous tau oligomers, whereas, at low ratios, PHF are formed. Both Ca^2+^ and Mg^2+^ binding to PHF favour the conversion into amorphous off-pathway aggregates. A neurofibrillary tangle is depicted in a representation at the bottom left corner of the figure. See text for details. Adapted from [30].

Tau is a disordered cytosolic protein involved in microtubule assembly and stability whose aggregation and toxic deposition are triggered by hyperphosphorylation. This results in the formation of intracellular tau paired helical filaments (PHF), which ultimately gather to form the characteristic neurofibrillary tangles (NFT) [70, 71], a process which is modulated by metal ions [30] (Figure 2). Zn^2+^ binds tau and promotes its hyperphosphorylation [72]; however, low concentrations of zinc induce fibril formation whereas high concentrations induce granular aggregates [73]. Fe^3+^ also binds to hyperphosphorylated tau and induces its aggregation [74, 75], mostly into PHF [75]; however, reduction to Fe^2+^ can reverse aggregation of tau [75]. Excess of iron is accumulated in NTF [76, 77] generating oxidative stress due to the Fenton reaction and perpetuating tau hyperphosphorylation [78]. The role of Cu^2+^ in tauopathies is controversial. Some studies suggest that tau binds Cu^2+^ [79], inhibiting its aggregation* in vitro* [80] while promoting tau hyperphosphorylation in hippocampal neurons [81]. Other studies however suggest that addition of copper-bis(thiosemicarbazone) complexes that increase intracellular copper in AD mice brains inhibits tau phosphorylation [82].

### 2.3. Amyloid-Beta Precursor Protein

Abnormal processing of the amyloid precursor protein leads to neurotoxic A*β* production. The proteolytic processing of APP is influenced by metal ions, by protein ligands, and by the APP oligomerization state. Cu^2+^ and Zn^2+^ promote APP expression [83–85] and possibly interfere with A*β* metabolism. Cu^2+^ enhances APP dimerization and increases extracellular release of A*β* [86]; yet, other studies suggest that high copper concentrations modulate APP processing leading to reduced A*β* production [87]. Interestingly, APP contains a copper binding domain and a site that favours Cu^+^ coordination, which has led to the suggestion that it could act as a neuronal metallotransporter [87]. Recent structural and biochemical studies have uncovered a high-affinity binding site within the E2 domain that binds competitively Cu^2+^ and Zn^2+^ at physiological concentrations [88]. Metal binding results in large conformational changes and in different structural states that regulate the function of APP and A*β* metabolism [89]. Indeed, APP can be a mediator of Cu neurotoxicity since it was shown that in primary neuronal cultures APP loaded with Cu^2+^ induces cell death [90]. This may possibly involve catalytic reduction of Cu^2+^ to Cu^+^ leading to an increase in oxidative stress in neurons [91]. The links between APP and metal metabolism are further emphasized by the interaction of APP with ferroportin, to promote iron export and its ferroxidase activity [92, 93]. APP ferroxidase activity is inhibited by Zn^2+^ binding contributing to Fe^2+^ accumulation in AD brains [92].

### 2.4. Presenilin-1

Presenilin-1 (PS-1) is a component of the *γ*-secretase multicomplex, responsible for the cleavage of APP. Presenilins have an activity as low-conductance passive ER Ca^2+^ leak channels which is independent of *γ*-secretase activity [94]. Overexpression of presenilin results in increased Ca^2+^ release whose levels are restored by *γ*-secretase inhibitors [95]. Mutations in presenilins as in familial AD forms result in downregulation of Ca^2+^ channels and Ca^2+^-dependent mitochondrial transport proteins, strengthening the relationship between Ca^2+^ homeostasis and presenilin [94, 96, 97]. A recent study based on the effects of metal chelators on *γ*-secretase suggests that Ca^2+^ and Mg^2+^ stabilize *γ*-secretase and enhance its activity [98].

### 2.5. Metallothionein 3

Metallothioneins are a family of ubiquitous proteins with metal binding properties and antioxidant activity [99]. Neuronal metallothionein 3 (MT3), which is involved in the transport and homeostasis of Zn^2+^ and Cu^2+^, plays an important role in several AD related pathways. MT3 is decreased in AD patients [100] and in Tg2576 mice [101], which can lead to aberrant neuritic sprouting [100]. Additionally, MT3 increases sAPP*α* (soluble amyloid precursor protein *α*) levels and reduces A*β* production [102], through an increase in ADAM10 (a disintegrin and metallopeptidase 10). ADAM10 is a protein responsible for the cleavage of APP-derived peptides and activation of the nonamyloidogenic pathway [103]. Mechanistically, it has been reported that the *β*-domain of MT3 interacts with A*β*, abolishing Cu^2+^ mediated aggregation [104, 105] and ROS production [104]. It has also been suggested that rapid metal exchange between Zn^2+^-MT3 and Cu^2+^-A*β* [106] or Zn^2+^ release by MT3 [107] promotes structural changes in A*β* aggregates. In agreement with this, in primary neuron cultures, MT3 inhibits the formation of toxic A*β* aggregates alleviating their neurotoxic effects [105, 108]. One possible mechanism for this effect may be related to the observed metal swapping between MT3 and soluble and aggregated A*β*, which abolishes the production of Cu-induced ROS [104, 109].

### 2.6. Zinc Transporter 3

Zinc transporter 3 (ZnT3) is a synaptic Zn^2+^ transporter responsible for loading zinc into presynaptic vesicles. This protein is highly expressed in the brains of AD transgenic mice, in which it colocalizes with amyloid plaques [110–112], where zinc is also found at high concentrations. Zinc sequestering within amyloid plaques has been suggested to provoke an imbalance in the cellular environment with concurrent effects on overall metal metabolism and protein homeostasis [35]. ZnT3 has been shown to decrease with aging and AD, contributing to the aggravation of zinc-mediated cognitive decline [113]. In the AD Tg2576 transgenic mouse model with a ZnT3 knockout, cerebral A*β* deposition was nearly abolished by the lack of synaptic Zn^2+^ [58, 59]. ZnT3 and other zinc transporters, such as ZnTs 1, 4, 5, 6, and 7, are also found upregulated in amyloid plaques of human AD brains near Zn^2+^ enriched terminals [60], revealing a cross talk between zinc induced amyloid plaques and zinc transporters. In ZnT3 knockout mice, the addition of metal chaperones results in restoration of expression of the synaptic proteins PSD-95, AMPAR, and NMDAR2b, due to the restitution of hippocampal zinc content [113].

### 2.7. ProSAP/Shank Scaffold Proteins

ProSAPs/Shanks are zinc-regulated multidomain proteins that are important scaffolding molecules of the postsynaptic density (PSD), a protein dense structure composed of both membranous and cytoplasmic proteins localized at the postsynaptic plasma membrane of excitatory synapses [114]. Deregulation of ProSAP/Shank has been reported in AD: in patients brains and in transgenic mice models, the accumulation of A*β* oligomers is accompanied by reduction of synaptic scaffold protein levels, such as Shank1 and ProSAP2/Shank3 [115], and disruption of the Homer1b and Shank1 scaffolds [116]. Interestingly, sequestration of Zn^2+^ by A*β* leads to less mature synapses by decreasing Shank1 protein levels at the postsynaptic density in hippocampal neurons [117]. Future studies will further elucidate the mechanistic cross-links between the presence of A*β*, zinc levels, and the scaffolding PSD proteins in the context of AD [118].

### 2.8. Ferritin

Ferritin is the major intracellular iron storage protein in the body. It has elevated levels in AD brain tissue [119–121] and is found in the vicinity of AD plaques [120], suggesting that ferritin trapped within the plaque inclusions may block the transport of iron between cells. The loss of integrity of hippocampus tissue of AD patients is linked with the increase of ferritin [122] and with a reduction of ferroportin protein levels [123]. Effectively, the impact of iron on AD outcomes is not fully explored but a recent longitudinal study has shown that ferritin is strongly associated with cerebrospinal fluid apolipoprotein E levels; in turn, ferritin is elevated by the Alzheimer's risk allele, APOE-*ε*4 [124]. This study speculates that the APOE-*ε*4 genotype raises the baseline iron load in the AD brain, lowering the threshold for iron-mediated neuronal loss, a hypothesis that remains to be experimentally addressed.

### 2.9. S100 Proteins

S100 proteins are a family of at least 21 different vertebrate-specific proteins with two Ca^2+^-binding EF-hand type sites and in some cases additional sites for Zn^2+^ and Cu^2+^ [125]. S100 proteins are part of the inflammatory response and a number of these proinflammatory cytokines (S100B, S100A6, S100A7, S100A1, S100A9, and S100A12) have been implicated in neurodegenerative disorders, such as AD.

S100B is a proinflammatory cytokine that triggers glial cell proliferation in a RAGE-dependent manner [141]. RAGE is an immunoglobulin-like cell surface receptor that is upregulated in AD and triggers the expression of proinflammatory cytokines and mediates A*β* transport across the blood-brain barrier [142–144]. At high micromolar concentrations, S100B promotes neuroinflammatory processes and neuronal apoptosis [145]. Increased expression of S100B by plaque-associated astrocytes in AD contributes to the appearance of dystrophic neurites overexpressing *β*APP in diffuse amyloid deposits [132]. Astrocytic overexpression of S100B is correlated with the degree of neurite pathology in A*β* aggregates and is induced by interleukin-1 (IL-1), which is secreted by activated microglia present in the plaques [146]. TNF*α*, a cytokine with high levels in AD, decreases S100B expression in astrocytes but increases its extracellular levels which can lead to RAGE activation [147]. Furthermore, studies demonstrated increased susceptibility to neuroinflammation and neuronal dysfunction after infusion of A*β* in transgenic mice overexpressing S100B [148]. Interestingly, S100B interacts with tau in a Zn^2+^ dependent fashion that could be responsible for neurite outgrowth [133]. Other studies, however, suggest that the S100B:tau interaction is mediated by Ca^2+^/calmodulin-dependent kinase II and results in the inhibition of tau phosphorylation [134].

S100A6, S100A9, and S100A12 also have consistently high levels in samples of AD patients [135, 149]. In particular, S100A9 is found near neuritic plaques [136, 137] and was found to coaggregate with A*β in vitro* and form toxic aggregates [136, 138]. Knockout of S100A9 in a transgenic mouse resulted in reduced A*β* levels in the brain and the animals presented an improved spatial reference memory [139]. In agreement with these observations, knockdown of S100A9 in the AD Tg2576 mice model reduced A*β* and APP C-terminal levels and decreased BACE activity [137]. Induction of S100A9 levels increased intracellular Ca^2+^ levels, which in turn upregulated secretion of the inflammatory cytokines IL-1*β* and TNF*α* [150]. On the opposite, expression of exogenous S100A7 in primary corticohippocampal neuron cultures derived from Tg2576 transgenic embryos inhibits the generation of A*β* and promotes the activity of *α*-secretase [140]. Interestingly, S100 proteins have been found to have amyloidogenic properties [151–155]. This feature, along with the high abundance of S100 proteins in protein deposits, their metal binding properties, dysregulation of Ca^2+^ signalling, and the high levels of Cu^2+^ and Zn^2+^ in the plaques, will certainly translate into the elucidation of new functions of S100 proteins in AD pathomechanisms.

## 3. Conclusion

Metal homeostasis and balance depend on a number of biochemical processes and proteins, many of which operate in the neuronal environment and in the extracellular synaptic space or at its interface. The biochemistry of this particular cellular moiety is deeply altered upon aging and under neurodegeneration, with wide changes in protein levels, signalling molecules, and metal ion concentrations. Changes in protein and metal ion homeostasis are hallmark features across amyloid-forming neurodegenerative diseases and as we have here overviewed, a number of proteins implicated in AD are directly regulated by metal-protein interactions; in some cases, metal ions are even directly involved as modulators of aggregation pathways. Uncovering the mechanistic details of this cross talk at the biochemical levels in respect to effects on synaptic protein networks, A*β* metabolism and intra- and extracellular protein aggregation in the context of concurrent affected processes such as oxidative stress and neuroinflammation are thus among the major challenges in modern molecular neurosciences.

## Figures and Tables

**Table 1:** Effect of metal ions on selected metal-binding proteins implicated in AD.

Protein	Metal	Effect	Model	Reference
A*β*	Cu^2+^	Modulates aggregation. Presence of Cu^2+^ in A*β* aggregates decreases toxicity; however, presence of Cu^2+^ in soluble A*β* accelerates cell death. Substoichiometric levels of Cu^2+^ render A*β* aggregates more toxic.	Synthetic A*β*, HEK cells, primary hippocampal cells, and PC12 cells	[49, 52–55, 57–59]
		Increases oxidative stress and neurotoxicity.	Synthetic A*β*, primary neuronal cells	[60, 63, 126, 127]
	Zn^2+^	Modulates aggregation. Zn^2+^ leads to less toxic A*β* aggregates.	Synthetic A*β*, HEK cells, and primary cortical cells	[49, 53, 54, 57]
	Fe^2+^	Modulates aggregation promoting the formation of annular protofibrils.	Synthetic A*β*	[49, 68, 69]
		Increases protein levels by disruption of APP processing.	Primary cortical neurons, APP/PS1 mice model, and HEK cells	[67]
		Increases oxidative stress.	M17 neuroblastoma cells, *Drosophila* model	[61, 62]

Tau	Cu^2+^	Modulates phosphorylation.	Tg-AD mice model, SH-SY5Y cells, and AD mice model	[81, 82]
		Modulates aggregation.	Peptide from tau first microtubule-binding repeat	[80]
	Zn^2+^	Induces phosphorylation through Zn^2+^ PP2A inhibition.	Rat brain slice cultures, primary neuronal cells	[72]
		Induces fibril formation via disulfide cross-linking.	Recombinant tau protein	[73]
	Fe^2+^	Modulates aggregation.	Recombinant tau protein, isolated hyperphosphorylated tau from human AD brain tissue	[74, 75]
		Induces imbalance in Cdk5/p25 function that causes a decrease in tau phosphorylation and an increase in oxidative stress.	Primary hippocampal cells	[78, 128]

APP	Cu^2+^	Increases APP expression levels and A*β* secretion. Promotes APP trafficking and its redistribution.	SH-SY5Y cells, polarized epithelial cells, MDCK-APP-cherry cells, primary cortical neurons, N2a cells, and APP/PS1 mouse model	[81, 83, 85, 86]
		Increases oxidative stress. Cu^2+^-metalated APP ectodomain promotes neuronal cell death.	Recombinant APP protein and mutants, primary neuronal cells	[90, 91]
	Zn^2+^	Inhibits ferroxidase activity.	Human brain tissue	[92]
		Increases APP expression levels and amyloidogenic cleavage that leads to accumulation of A*β*.	SH-SY5Y cells, APP/PS1 mice model	[84]
	Fe^2+^	APP interacts with ferroportin and promotes iron export.	Human brain tissue, HEK293 cells	[92, 93]

Presenilin	Ca^2+^	Overexpression of PS1 decreases Ca^2+^ release from ER and downregulates Ca^2+^-dependent mitochondrial transport proteins. Expression of PPS1 M146V causes inhibition of Ca^2+^ channels.	HEK293 cells, human brain tissue, SH-SY5Y cells, SK-N-SH cells, and APPswe/PS1dE9 mice model	[95–97]

MT3	Cu^2+^ Zn^2+^	Decreases protein levels.	Human brain tissue, Tg2576 mouse model	[100, 101]
		MT3 interacts with A*β* inhibiting/modulating A*β* aggregation and cytotoxicity.	Recombinant MT3 protein and synthetic A*β*, SH-SY5Y cells, primary cortical cells, and Tg2576 mouse model	[104–107]
		Metal swapping between MT3 and A*β* lowers ROS production and decreases neurotoxicity.	Recombinant MT3 protein and synthetic A*β*, SH-SY5Y cells	[109]
		MT3 increases sAPP*α* levels and reduces A*β* production.	N2a Swedish APP cells	[102]

ZnTs	Zn^2+^	Increases expression levels and colocalization with amyloid plaques.	APP/PS1 mouse model, human brain tissue	[110–112, 129]

ProSAP/Shank scaffold proteins	Zn^2+^	Zn^2+^ sequestering by A*β* decreases Shank1 and ProSAP27Shank3 protein levels and promotes synapse loss by disruption of Homer1b and Shank1 scaffold.	Primary hippocampal cells, human brain tissue, and Cos7 cells	[115–117]
	Ca^2+^	Homers 2 and 3 interact with APP inhibiting APP processing and consequently reducing A*β* secretion.	HEK293 cells, C57/Black6 mouse model	[130, 131]

Ferritin	Fe^2+^	Increases protein levels. Present within and around amyloid plaques and neurofibrillary tangles.	Human brain tissue	[119–121]

S100B	Ca^2+^	Increased expression of S100B contributes to overexpressing *β*APP in diffuse amyloid deposits.	Primary neuron cells	[132]
	Zn^2+^ Ca^2+^	S100B interacts with tau resulting in the inhibition of tau phosphorylation via Ca^2+^/calmodulin-dependent kinase II.	Bovine S100B, SH-SY5Y cells	[133, 134]

S100A9	Ca^2+^	Increases protein levels. Present near amyloid plaques. Interacts with A*β* *In vitro* and forms linear and annular aggregates. Knockout of the S100A9 gene reduces neuropathology due to reduced A*β* and APP C-terminal levels.	Human brain tissues, Tg2576 mice model, SH-SY5Y cells, and S100A9 recombinant protein	[135–139]

S100A7	Ca^2+^	Expression of exogenous S100A7 inhibits A*β* production and promotes *α*-secretase activity.	Primary corticohippocampal cells	[140]
